# A 40‐year‐old man with a rapidly growing intrascrotal tumor in the fibroma–thecoma group

**DOI:** 10.1002/iju5.12430

**Published:** 2022-03-17

**Authors:** Tsutomu Anraku, Hideki Hashidate, Asa Nakahara, Tomoyuki Imai, Yoshiaki Kawakami

**Affiliations:** ^1^ Department of Urology Niigata City General Hospital Niigata Japan; ^2^ Department of Pathology Niigata City General Hospital Niigata Japan

**Keywords:** fibrothecoma, intrascrotal tumor, orchiectomy, paratesticular tumor, tumors in the fibroma–thecoma group

## Abstract

**Introduction:**

Tumors in the fibroma–thecoma group are benign tumors, typically found in the ovaries of postmenopausal women and occasionally develop in the testes. These tumors are mostly treated with radical orchiectomy because preoperative diagnosis confirming the benign nature is difficult.

**Case presentation:**

A 40‐year‐old man was incidentally pointed out to have a right intrascrotal mass, measuring approximately 10 cm on computed tomography. Malignant testicular tumor was suspected based on the location and size of the tumor. The patient underwent right radical orchiectomy. Histologically, the tumor had no evidence of malignancy, and the diagnosis of tumors in the fibroma–thecoma group was made. The patient had no recurrence 8 months after surgery.

**Conclusion:**

Intrascrotal tumors in the fibroma–thecoma group are rare benign tumors and mostly treated with radical orchiectomy due to concerns about malignancies. Further investigation is needed for accurate preoperative diagnosis, and we should be aware of these rare tumors.

**Abbreviations & Acronyms**
AFP: α‐fetoprotein
BCL2: B‐cell/CLL lymphoma 2
CK AE1/AE3: Cytokeratin AE1/AE3
CT: Computed Tomography
HCG: Human chorionic gonadotropin
KIT: CD117
LDH: Lactate dehydrogenase
MRI: Magnetic Resonance Imaging
SMA: Smooth muscle actin
STAT6: Signal transducer and activator of transcription 6

**Keynote massage**

Intrascrotal tumors in the fibroma–thecoma group are rare benign tumors. Radical orchiectomy is most often performed, and the prognosis is excellent.

## Introduction

Tumors in the fibroma–thecoma group are benign tumors, typically found in the ovaries of postmenopausal women. They are classified as sex cord–stromal tumors and account for approximately 4–7% of all gonadal neoplasms. Surgical resection is the primary treatment and is usually curative. In men, tumors in the fibroma–thecoma group occasionally develop in the testes. These tumors are exceedingly rare and typically measure 0.5–8 cm (mean, 2 cm).^1^ We report on a rapidly growing tumor in the fibroma–thecoma group, derived from the testicular tunica albuginea, in a 40‐year‐old man.

## Case presentation

**Fig. 1:**
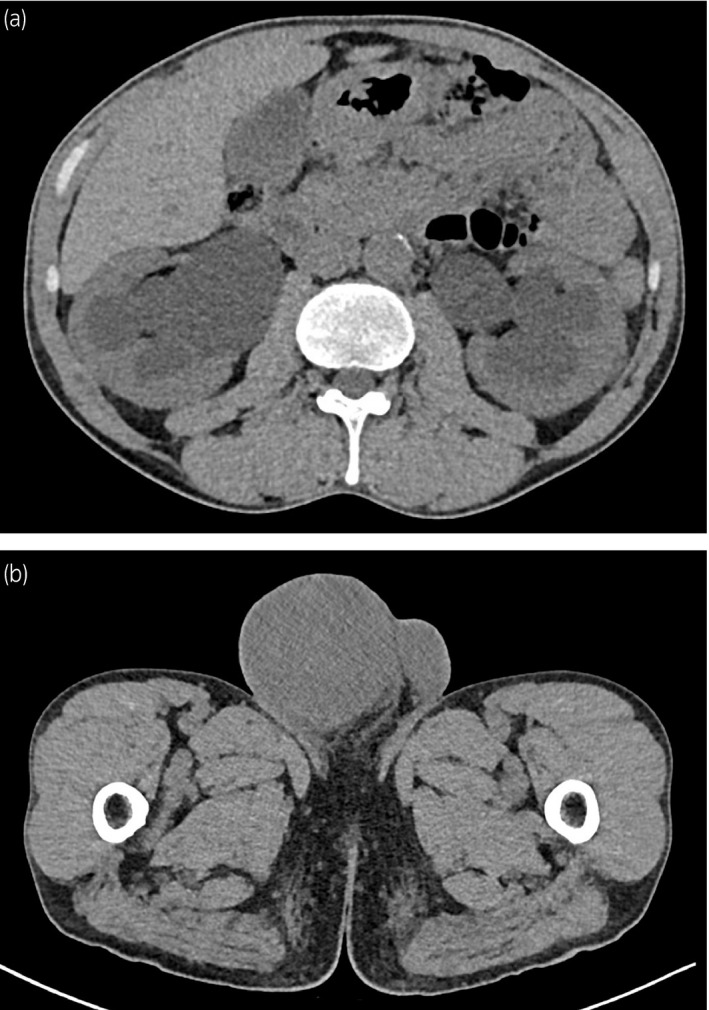
(a) CT scan showing bilateral hydronephrosis worse than a year ago. (b) CT scan showing an about 10 cm right intrascrotal mass.

**Fig. 2:**
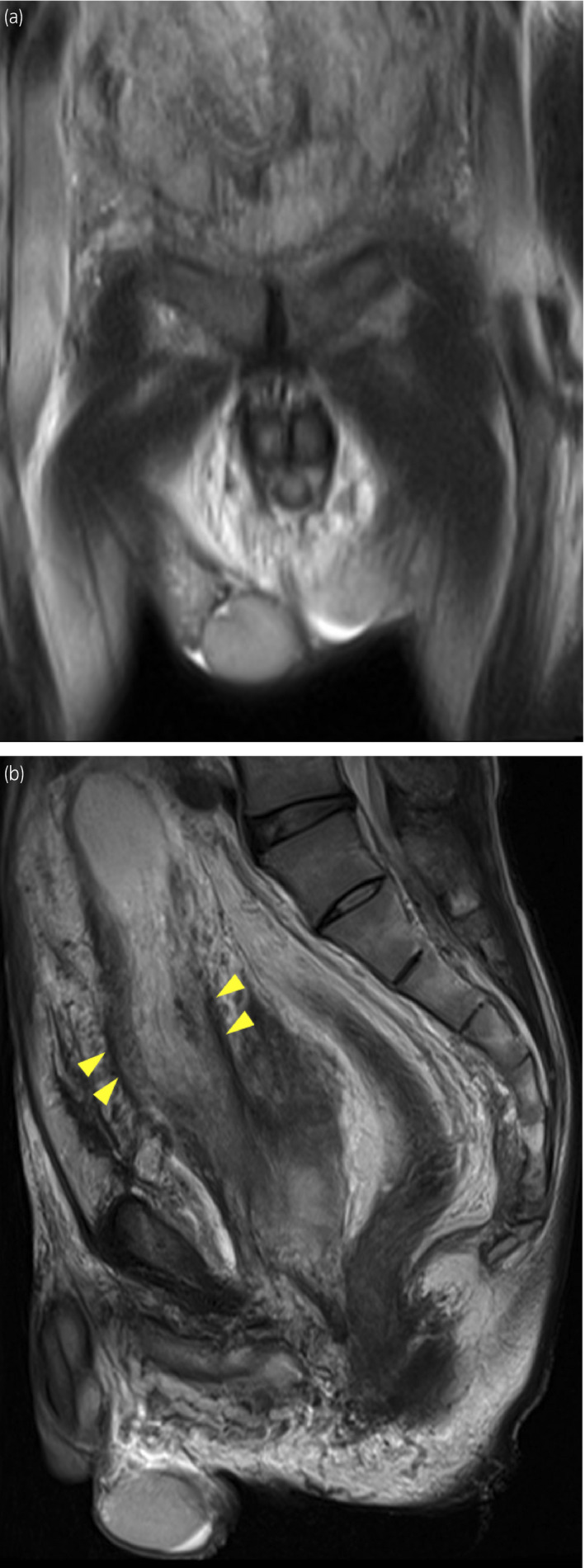
(a) MRI a year ago showing normal right testis. (b) MRI a year ago showing a thickening and poor dilation of the bladder wall from the neck to the trigone. The thickened bladder wall is indicated by arrowhead. The size of the right testis is 3.3 × 2.4 cm.

A 40‐year‐old man was incidentally pointed out to have a right intrascrotal mass measuring approximately 10 cm on computed tomography taken to follow up on bilateral hydronephrosis (Fig. 1). A year ago, he was diagnosed with mild bilateral hydronephrosis with unexplained obstruction of the distal ureter. Magnetic resonance imaging performed at that time revealed a thickening and poor dilation of the bladder wall from the neck to the trigone, suggesting a congenital anomaly. There was no reported abnormality in the right testis (Fig. 2).

Scrotal ultrasonography showed a hypoechoic solid mass (Figure S1). Testicular tumor markers (HCG, AFP, and LDH) were within normal limits. Based on the location and size of the tumor, malignant testicular tumor was suspected. The patient underwent right radical orchiectomy. At the same time, a left ureteral stent insertion was performed because bilateral hydronephrosis and renal dysfunction gradually progressed. A right ureteral stent insertion was also attempted, but was unsuccessful due to the severe flexion of the ureter. Renal function of the patients did not improve postoperatively .

**Fig. 3:**
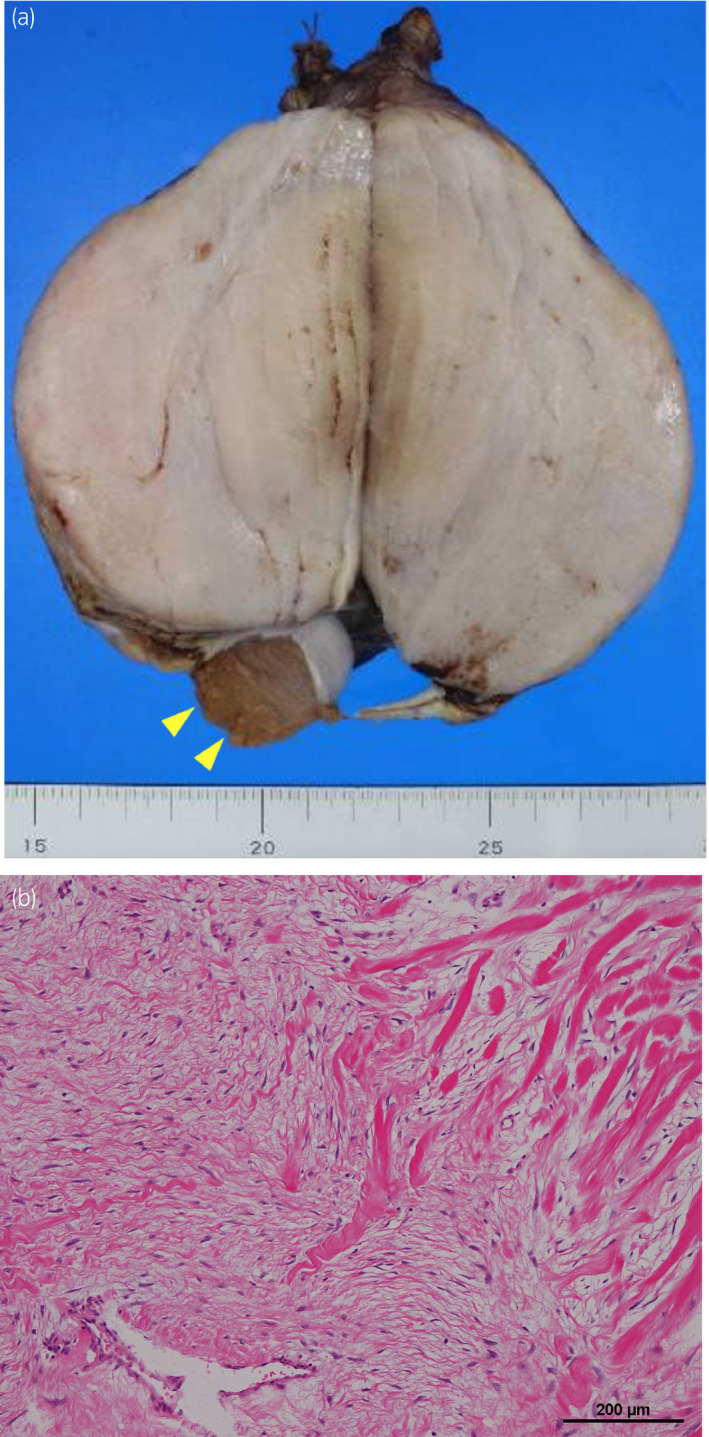
(a) Gross appearance of the tumor (12 × 7×5 cm). It was well circumscribed solid mass with no continuity with the testis. The normal right testis is indicated by arrowhead. (b) High power image showing sparsely arranged spindle cells with collagen deposits in the stroma.

Macroscopically, the tumor was a solid, elastic, yellow‐grayish, hard mass measuring 12 × 7×5 cm and continuous with the testicular tunica albuginea but not with the testis (Fig. 3a and S2). Microscopically, the tumor showed expansive growth without a fibrous capsule. Spindle cells were sparsely arranged, and collagen deposits were apparent in the stroma (Fig. 3b and S3). There was no invasion of the lymphovascular system, tunica vaginalis, or spermatic cord. Mitosis was very rare, with <1 per 50 high‐power fields. Immunohistochemistry showed positive staining for vimentin and BCL2, weak staining for SMA, and negative staining for inhibin‐α, calretinin, CK AE1/AE3, STAT6, KIT, Melan‐A, S‐100, desmin, and CD34 expressions. The Ki‐67 index was <1%. These findings suggested a diagnosis of a tumor in the fibroma–thecoma group. There were no malignant findings, and the margins were negative. The patient has no recurrence 8 months after surgery.

## Discussion

Although most intrascrotal masses are testicular tumors, there is a subset of extratesticular tumors arising from paratesticular tissues, including the spermatic cord, testicular tunics, epididymis, and vestigial remnants. Paratesticular tumors are rare and account for approximately 5% of intrascrotal neoplasms. Of these, approximately 75% arise from the spermatic cord.^2^ It is estimated that 70% of these tumors are benign.^3^ The most common paratesticular benign tumors are lipomas, adenomatoid tumors, and leiomyomas.^3^ Paratesticular fibromas are rare.

In 1997, Jones et al.^4^ classified benign intrascrotal fibromas into fibromas of gonadal stromal origin and fibromas of the testicular tunics. The former are testicular fibromas, which are homologous to the ovarian fibroma–thecoma group in women; the latter are paratesticular fibromas. The current WHO classification does not distinguish between testicular and paratesticular fibromas; they are collectively classified as tumors in the fibroma–thecoma group.^1^

Typically, fibromas of the testicular tunics are well‐circumscribed, white‐tan, or yellow. Microscopically, tumors are of low‐to‐moderate cellularity and are generally composed of uniform, randomly arranged spindle cells. The stroma is myxoid or collagenous, and inflammatory infiltrates are absent. Mitotic activity, nuclear atypicality, and necrosis are not usually seen.^4^

In our case, the tumor was continuous with the testicular tunica albuginea but not with the testis. Although the possibility of testicular stromal origin cannot be completely ruled out, the negative expression of inhibin‐α and calretinin might exclude tumors of gonadal stromal origin. The differential diagnosis of this tumor includes solitary fibrous tumor and other sex cord‐stromal tumors.^5^ In our case, the HE staining findings of the tumor are histologically most consistent with tumors in the fibroma–thecoma group in the first place.^1^ Immunohistochemical findings can also aid in differential diagnosis. Solitary fibrous tumor is usually positive for STAT6, and other sex cord‐stromal tumors are usually positive for some of the following: inhibin‐α, calretinin, S‐100, and Melan‐A. In addition, positive staining for BCL2 supports that the tumor in our case was tumor in the fibroma–thecoma group rather than the differential diagnosis described above. The tumor in our case grew rapidly to 12 cm, the second‐largest ever reported,^6^ despite its low mitotic activity, as indicated by rare mitosis and low Ki‐67 index. The reason for the discrepancy between tumor growth rate and pathological findings is unclear. The bilateral hydronephrosis and renal dysfunction appeared to be unrelated to the tumor.

Paratesticular tumors in the fibroma–thecoma group are mostly treated with radical orchiectomy, and there have been no recurrent cases reported.^4^ In this case as well, radical orchiectomy was performed because malignancy was clinically suspected. However, this case of a tumor in the fibroma–thecoma group shows that even large and rapidly growing intrascrotal masses can be benign tumors. Further investigation is needed for accurate preoperative diagnosis, because a preoperative diagnosis confirming the benign nature of intrascrotal mass based on imaging findings may enable testicular preservation surgery.^7^

## Author Contributions

Tsutomu Anraku: Conceptualization; Writing – original draft. Hideki Hashidate: Investigation; Supervision; Validation. Asa Nakahara: Validation; Visualization. Tomoyuki Imai: Conceptualization; Supervision. Yoshiaki Kawakami: Supervision.

## Conflict of interest

The authors declare no conflict of interest.

## Approval of the research protocol by an Institutional Reviewer Board and the approval number

Not applicable.

## Informed consent

Written informed consent was obtained from the patient for the publication of this case report.

## Registry and the Registration No. of the study/trial

Not applicable.

## Funding source

This research did not receive any specific grant from funding agencies in the public, commercial, or not‐for‐profit sectors.

## Supporting information


**Figure S1**. Scrotal ultrasonography showed a hypoechoic solid mass.

**Figure S2**. Gross appearance of the tumor before formalin fixation.

**Figure S3**. Microscopic appearance of the tumor tissue composed of spindle‐shaped cells.
