# Supplementary figures and images for: A 40‐year‐old man with a rapidly growing intrascrotal tumor in the fibroma–thecoma group
Source: IJU Case Rep. 2022 Mar 17;5(3):175–8. doi: 10.1002/iju5.12430 (PMC9057753; doi:10.1002/iju5.12430)

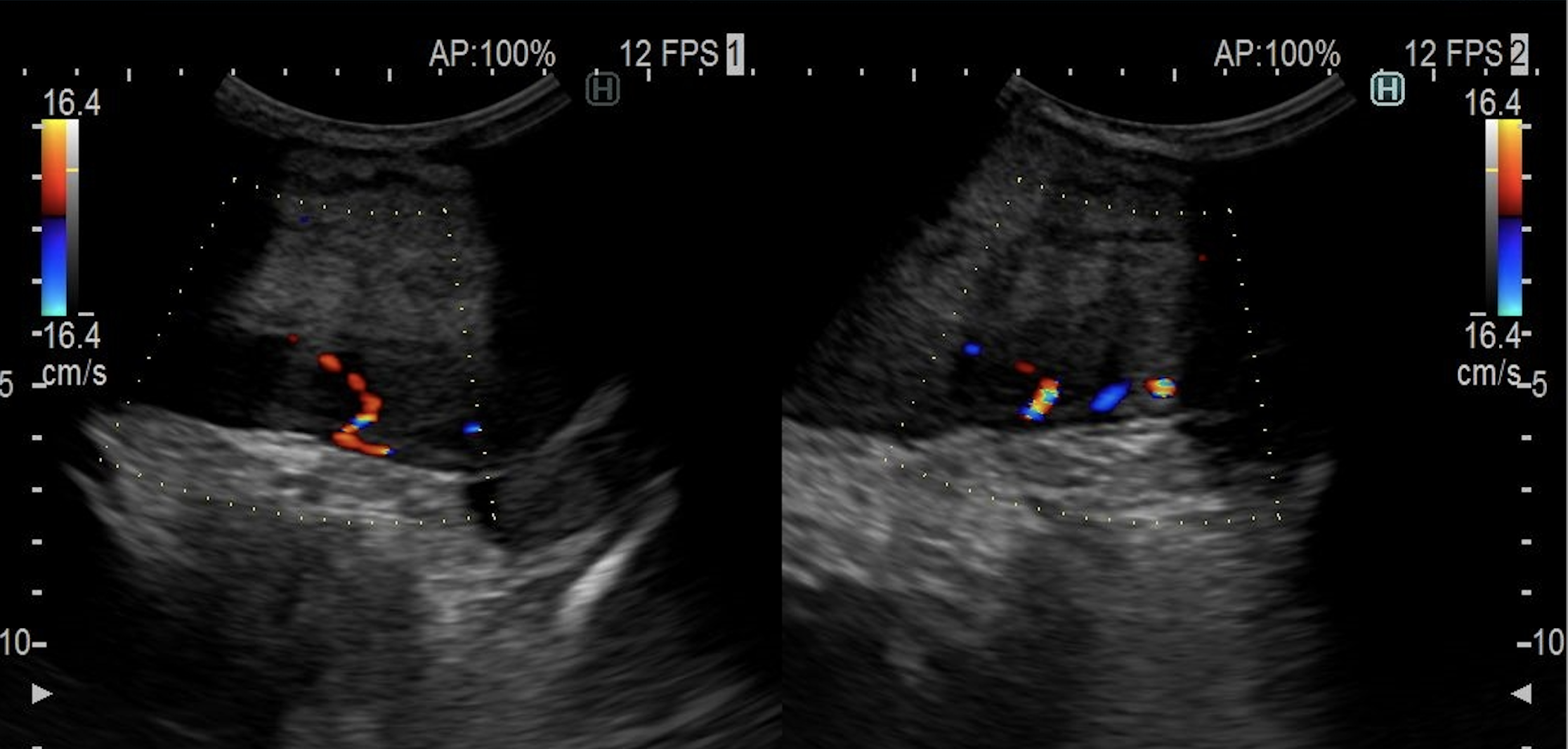

Supplement: Supplementary file 1 — Figure S1. Scrotal ultrasonography showed a hypoechoic solid mass. [file IJU5-5-175-s001.tiff]

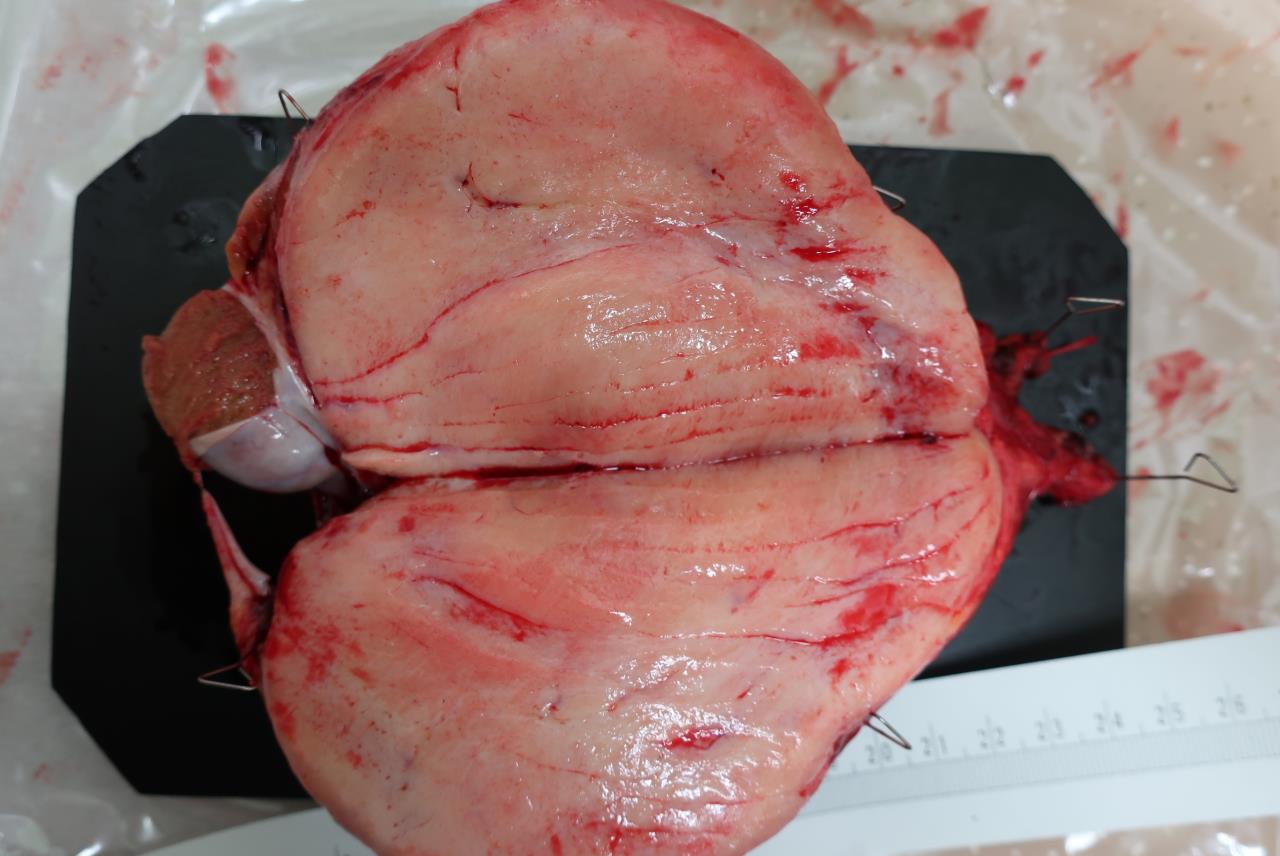

Supplement: Supplementary file 2 — Figure S2. Gross appearance of the tumor before formalin fixation. [file IJU5-5-175-s002.jpg]

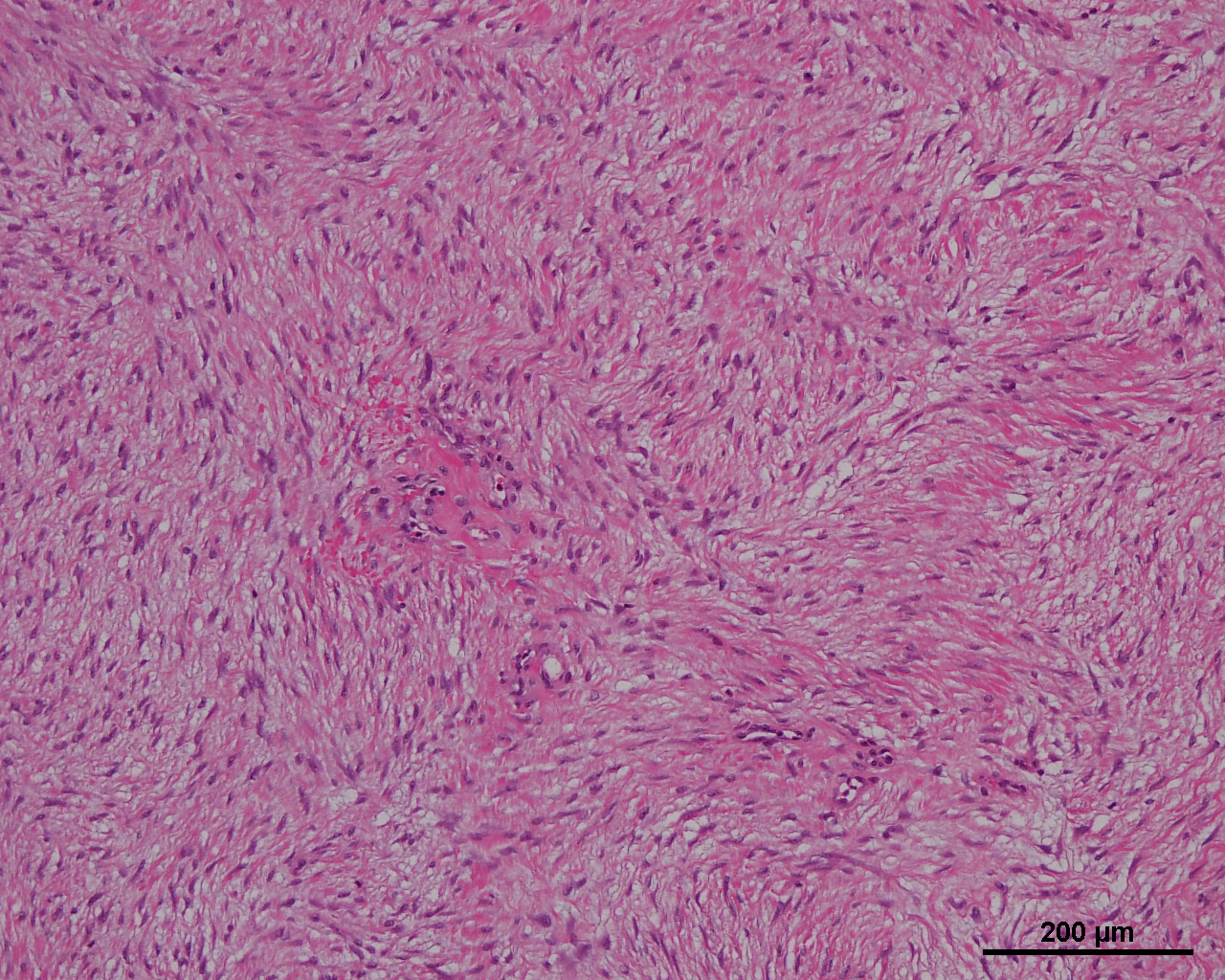

Supplement: Supplementary file 3 — Figure S3. Microscopic appearance of the tumor tissue composed of spindle‐shaped cells. [file IJU5-5-175-s003.jpg]
